# In support of descriptive studies; relevance to translational research

**DOI:** 10.1186/1479-5876-5-21

**Published:** 2007-05-02

**Authors:** Francesco M Marincola

**Affiliations:** 1 Infectious Disease and Immunogenetics Section, Department of Transfusion Medicine, Clinical Center, National Institutes of Health, Bethesda, MD 20892, USA

## Abstract

The contemporary scientific establishment equates hypothesis testing to good science. This stance bypasses the preliminary need to identify a worthwhile hypothesis through rigorous observation of natural processes. If alleviation of human suffering is claimed as the goal of a scientific undertaking, it would be unfair to test a hypothesis whose relevance to human disease has not been satisfactorily proven. Here, we argue that descriptive investigations based on direct human observation should be highly valued and regarded essential for the selection of worthwhile hypotheses while the pursuit of costly scientific investigations without such evidence is a desecration of the cause upon which biomedical research is grounded.

*There are good things so in the tide pools and interesting thoughts to be generated from the seeing. Every new eye applied to the peephole which looks out at the world may fish in some new beauty and some new pattern, and the world of the human mind must be enriched by such fishing*.

*John Steinbeck – Foreword to the Third Edition of Ed Ricketts' "Tides"*.

## Descriptive studies will increase the efficiency of translational research

It is surprising how often a manuscript is dismissed by reviewers as "just descriptive", regardless of the novelty of the reported observation. On the other hand, we have not once received a negative comment on a "mechanistic" study, even if it lacks proof of the validity of the experimental model and its relevance to human disease. Such studies are automatically given the benefit of the doubt based on predictable rationalizations vaguely offered in the introductory paragraphs. As a consequence, innumerable conflicting results are published, each one a reflection of its own experimental bias. For example, in animal models, Interleukin-23 can either promote or hamper cancer growth [1-6]; yet, information about its bio-availability in human cancers and its modality of expression, information that can potentially provide insight into the interpretation of such models, is limited. As previously argued [7-13], translational research is a two way road with much to be learned from the unbiased study of human beings and if a claim of relevance to human suffering is placed, compelling evidence should be provided.

Should studies that claim mechanistic explanations of human pathology be required to provide descriptive evidence of their applicability to humans? Should such evidence be considered the horse pulling the cart, or can the cart sometimes lead the horse? When is it appropriate to search for mechanistic explanations and apply hypothesis testing to the study of human disease? If one were to serendipitously discover a drug that cures 100% of human cancers, would we disregard it because we do not know its mechanism of action? Would indeed the mechanism matter? Shouldn't scientists focus on other medical problems if truly interested in human welfare? Obviously, luck alone is not likely to lead to big breakthroughs and the chance of identifying a successful drug would be slim using a random approach. A more reasonable strategy requires the formulation of hypotheses based on available knowledge. The problem occurs when the hypothesis causes the investigator to drift away from human observation. When an investigator allows the hypothesis to restrain his or her power of observation, hypothesis-driven research can yield surprising conclusions. For example, at a recent conference on melanoma, five speakers presented back-to-back talks in which each provided convincing evidence that her or his protein was "the orchestrator" of melanoma metastases and, therefore, the preferred target of future therapies. Nobody in the audience, taken by the elegance of the presentations, noted the contradiction in these serial claims of five different sources, each exclusively providing a mechanistic explanation for the same phenomenon. Strikingly, descriptive confirmation about the actual expression of these proteins in human melanoma was neither provided nor requested. Why should we risk tarnishing the beauty of our theories with facts?

Basing hypotheses on preliminary experimental observation is dictated by the scientific method to which most of us subscribe. Sir Francis Bacon would agree that the quality of a hypothesis depends on the quality and relevance of the facts upon which it is based. The Scientific Revolution has been driven by induction that draws knowledge from the natural world through experimentation, observation, and testing of hypotheses. It is true that facts can only be confirmed by experimentation that is reproducible and testable; to achieve this goal, experiments are controlled, ideally, by testing one variable at the time. However, hypothesis testing is not meant to validate the foundation upon which the hypothesis itself is based. Instead, experimentation and observation should lead to an accurate description of the facts upon which we could base a relevant hypothesis. Kepler's description of the laws of planetarian motion came much before a mechanistic explanation could be offered by Newton's theory of universal gravitation. Kepler interpreted his findings through a mixture of scientific and religious arguments. However, his accurate and predictive descriptions provided the coordinates for the formulation of Newton's theory. Should Kepler's work have been dismissed as simply descriptive?

*Two botanists heard that in a village in the Country grew Blue Chrysanthemums. For a whole summer they tried hard to find them and as they were to give up they saw a girl, Klara, the village simpleton, carrying bouquets of Blue Chrysanthemums. On the day after, they followed the girl and saw a blue patch in a meadow they had not previously violated because of a posted "No Trespassing" sign. But the girl who could not read happily wandered into the field and found the Blue Chrysanthemums*.

Karel Capek – Short Novels

An observational approach is important also because it is unbiased. Sometimes, a simple solution is missed because we fail to observe the surrounding world with an open eye as pre-conceived ideas may stir our thinking away from novel pastures. Disrespect for observation is a recurring theme in history. Don Quixotes thrived for centuries chasing spectacular windmills and ignoring those Sancho Panchas who may confuse them with facts. In post-revolutionary France, the empirical method was born of the desire to upset institutional privileges, and with this fervor the modern discipline of surgery gained momentum. At the time, surgery wedded the medical discipline to the human body, violating the traditional teaching of medicine in Latin, in which philosophy and religion were dominant to the natural observation of cadaver dissection or patient examination. Observation was considered vulgar and irrelevant and logical thinking *de rigueur*. William Harvey's discovery that blood circulates was dismissed, and those who advocated and practiced direct human observation were not allowed to hold positions as professors, but could only be appointed as "demonstrators". It took a century for visual analysis to take over theoretical assumptions and for anatomy atlases to ratify the basis of human physiology [14].

## Where are we now?

Perhaps we should think in terms of theories and hypotheses only being useful when the potential for observation alone has been exhausted. Direct observation and discovery-driven approaches should be sought first. With the advent of modern high-throughput tools, existing theories based on oligo-thematic observations might be shaken. For example, global transcript analysis of melanoma metastases demonstrates that the administration of high dose interleukin-2 to cancer patients does not induce migration of T cells within the tumor microenvironment [15] as previously hypothesized to explain the observation that their disappearance from the circulation was associated with increased frequency of tumor regression [16]. In contrast, an unbiased discovery-driven approach demonstrated that interleukin-2 activates innate immune mechanisms that had not been suspected before and could not have been hypothesized based on available knowledge. This unprecedented opportunity to reevaluate the basis of human disease through high-throughput observation cannot be dismissed.

Yet, research funding practice strongly favors "hypothesis-driven" proposals, disregarding the biases that the minimization of experimental variability might introduce. Hypothesis testing is the conclusive step that validates a scientific observation but it does not support its relevance claim. Whether a hypothesis is relevant to a particular endeavor or not can only be tested within the studied entity; it cannot be extrapolated through artificial experimental models. Hypothesis testing aims to validate the reproducibility and specificity of observations that may explain a phenomenon. This approach is not necessarily efficient, and hypotheses may often be wrong or irrelevant if insufficient care is taken to collect facts to support it. In times of abundance, efficiency may not be the highest priority, and scientists might have the chance to indulge the luxury of speculative adventures in the world of the unknown. But in these times of restricted funding opportunity, it behooves us to select our scientific challenges parsimoniously by constantly confronting our intuitions with the reality of human pathology. Consider this theoretical example: two scientists want to identify the cause of a recurring noise on the other side of the wall. The first provides reasonable hypotheses and proposes an elaborate experimental model to reproduce the sound, implying a mechanistic relationship between the experimental and actual phenomena. Such a proposal would be highly praised by her/his peers because of the ingenuity of the doctrine applied for the solution of a formidable challenge. The second scientist proposes to buy the key that opens the door across the wall and look for what causes the noise. The latter would be considered demeaning to the scientific enterprise; over simplistic, unsophisticated, discovery-driven, lacking a true hypothesis and would therefore not be funded. But, in case common sense ought to apply to scientific thinking, we should turn this reality on its head, and demand that no grants be funded without supportive evidence that prior observational tools have been exhausted and the existence of a key and a door has been sufficiently sought and excluded. Similar requirements could be applied for the retrospective judgment of mechanistic, hypothesis-driven studies submitted for publication.

The *Journal of Translational Medicine *welcomes the submission of high quality, purely descriptive studies that provide novel information relevant to human disease. Mechanistic studies are also welcome. We hope that this assortment may facilitate a shift of scientific investigation toward human relevance. The discovery-driven approach is often derided as a "fishing expedition". This unwarranted lack of respect for piscatorial challenges inspires us to refer cynics to Steinbeck's "On Fishing", where the virtues of this ancient and noble art and its value in different cultures are eloquently defended [17]. To the fishermen, we wish good luck and eagerly await publication of their next big catch in the *Journal of Translational Medicine*.
